# Protein Glycosylation and Its Role in Current Immunotherapeutic Strategies

**DOI:** 10.3390/cancers18152471

**Published:** 2026-07-31

**Authors:** Marco Agostini, Pietro Traldi, Mahmoud Hamdan

**Affiliations:** Istituto di Ricerca Pediatrica Città della Speranza, Corso Stati Uniti 4, 35100 Padova, Italy; m.agostini@unipd.it (M.A.); mhglaxo@gmail.com (M.H.)

**Keywords:** protein post-translational modifications, cytokine release syndrome, checkpoints, CAR T cell therapy, mass-spectrometry-based proteomics

## Abstract

Proteins and associated post-translational modifications are receiving deserved attention in the search for immune therapies to combat a long list of diseases, including a number of fatal forms of cancer. Such attention has been fueled by a number of clinical results generated by numerous clinical trials, together with datasets generated by academic research. The title of this review is based on a couple of considerations: most, if not all, researched immune checkpoints are proteins, each of which can experience one or more post-translational modifications (PTMs). It is also known that among the main functions of post-translational modifications is their direct impact on protein localization. Given that most activities of known checkpoints are performed on the surface of the host cells, post-translational modifications are bound to influence the role of these proteins, both as drivers of various diseases and as therapeutic targets. The second consideration concerns another class of proteins, which is responsible for a severe toxic reaction in the immune system following treatment with immune cell inhibitors, a reaction known as cytokine release syndrome (CRS). Cytokines are low-molecular-weight proteins, among which the key members are interleukin-1 (IL-1), interleukin-6 (IL-6), and interferon γ (IFN-γ). A number of clinical trials have shown that the symptoms of CRS toxicities are frequently accompanied by elevated levels of cytokines, including IL-6 and IFN-γ. The recent literature suggests that we still need to know more about the biology of these proteins and the type of modifications that these key members of cytokines can experience. Such additional knowledge may contribute to more effective and safer immune cell therapy. The contribution of mass-spectrometry-based proteomics to the investigation of PTMs associated with immune checkpoints and cytokines is discussed.

## Simple Summary

This text discusses the impact of protein post-translational modifications (PTMs), specifically glycosylation, on ongoing immunotherapeutic strategies. The text focuses on the role of such modifications in the regulation of some immune checkpoints and cytokines and how such regulation influences the choice of potential therapeutic targets for cancer immunotherapy. The authors underline the key role of mass-spectrometry-based proteomics in the analysis of these modifications.

## 1. Introduction

Post-translational modifications (PTMs), together with alternative splicing of genes, are mainly responsible for the diversity and complexity of the human proteome. The number of protein-coding genes in the human genome has been established at approximately 20,000–25,000 [1]; yet, the current literature demonstrates that this unexpectedly low number of human genes is capable of expressing millions of proteins [2]. A good part of this impressive number of gene products is due to various PTMs, while most of these products are due to alternative splicing of mRNA transcripts, which enables the same gene to generate varied protein isoforms of different lengths. The interaction between proteins and other biomolecules is another source for proteome complexity. A number of immune checkpoints are known to experience various forms of PTMs. The most familiar and most studied among these modifications are glycosylation, phosphorylation, and ubiquitination. These three modifications make up part of a much larger family, which contains over 650 known modifications [3]. The complexity of both the chemistry and the biology behind protein modifications has been described in a number of recent works [4,5,6,7]. The approval of immune checkpoint inhibitors (ICIs) almost 15 years ago for the treatment of unresectable advanced melanoma has been justly described as a new era in the field of immune therapy [8,9]. However, years after the launch of these inhibitors, various clinical trials have revealed that a disappointingly low percentage of the treated patients demonstrated a substantial therapeutic benefit; while the remaining majority of patients could develop irreversible resistance and/or serious immune-related adverse events (irAEs) [10,11], some of these high-grade adverse events often lead to an interruption of the ICI therapy. These limitations have sparked intensive research activities to discover and develop effective predictive biomarkers capable of accurate identification of the patients who are likely to get tangible therapeutic benefit without exposing the same patients to serious irAEs. The same limitations have also encouraged the search for novel therapeutic strategies. One of these novel strategies is based on targeting post-translationally modified immune checkpoints. N-glycosylation of PD-L1 can be considered a representative example of how certain PTMs can modulate the efficacy of immune checkpoint inhibitors. Despite its limitations as a predictive biomarker, the PD-L1 level of expression on cancerous tissues remains one of the main predictive biomarkers in response to ICI therapy. This level of expression is normally assessed by using immunohistochemical (IHC) staining, a number of studies have demonstrated that such assessment can be negatively influenced by glycosylated PD-L1. To improve the reliability and efficiency of this biomarker, a de-glycosylation protocol had to be applied before IHC measurements [12,13,14].

Cytokines are small, secreted proteins with relatively low molecular weights (~5–25 Da). These proteins perform various important functions, including cell-to-cell communication, participation in various immune responses, and stimulation of cell migration to inflamed and trauma sites. Some of these proteins are also known to be involved in signaling for cell survival, proliferation, and differentiation [15,16,17].

The relatively recent adoption of cellular-based immunotherapies to treat various fatal cancers is considered one of the success stories in the world of oncology. This therapy demonstrated some success in the treatment of various forms of blood cancers, but not for solid tumors. The rapid success of CAR T cellular therapy was the direct result of combined advancement in the areas of tumor immunology, genetic engineering, and unprecedented development in cell manufacturing [18,19]. This success, however, has been strongly slowed down by the adverse events that accompany this form of therapy. Elevated expression of various cytokines is responsible for a serious adverse event known as cytokine release syndrome (CRS), which manifests during CAR T therapy. This adverse event involves the rapid and excessive release of cytokines into the blood stream, causing high fever, breathing difficulties, and, in serious cases, multi-organ failure.

### 1.1. CAR T Cell Therapy and Glycosylation

CAR T cell therapy has demonstrated reasonable success in the treatment of various types of blood cancer. The failure of the same technique to realize similar success against solid tumors is generally attributed to the scarcity of tumor-specific antigen expression and the heterogeneity of the cellular population within a complex tumor microenvironment (TME). Glycoproteins expressed by tumor cells and associated stromal cells represent attractive targets of CAR T therapy. Furthermore, alterations in the glycosylation pattern within the TME can help cancer cells to escape immune surveillance. Various studies have demonstrated that targeting glycosylated proteins within the TME has the potential of improving anti-tumor responses. It is argued that glycosylation exerts its biological functions through the complexation of different glycans with lectins, a specific family of proteins [20,21]. Targeting glycosylated immune checkpoints with CAR T cell therapy has highly promising therapeutic potential, particularly for the treatment of some solid tumors. That said, a number of challenges have to be addressed before such potential can be translated into clinical practice. One of these challenges is how to enhance the efficacy of CAR T therapy in the treatment of solid tumors. Addressing such challenge, a number of factors have to be considered. It is known that most solid tumors originate from normal epithelial tissue, where the identification of tumor-specific antigens not expressed on healthy tissues has proven difficult [22]. In contrast to blood cancers, where easy circulation facilitates ample interaction with CAR T therapy, in solid tumors, the same therapeutic cells have to infiltrate and expand in discrete solid tumor sites. These factors are further aggravated by a hostile TME, where various mechanisms of inhibiting both physiologic T cell and CAR T cell infiltration of the targeted solid tumors can take place.

#### Some Methods to Detect Protein Post-Translational Modifications

Currently, there is a limited number of analytical methods that can be routinely used to detect and, above all, identify the site(s) of modification. Tandem mass spectrometry (MS/MS) coupled with liquid chromatography (LC) remains the most popular tool for such analysis [23,24]. Western blotting is another technique, commonly used in protein analysis, including post-translationally modified proteins. In this method, proteins are separated using gel electrophoresis, followed by transfer to a membrane to be examined by antibodies specific to the modification under examination. An enzyme-linked immunosorbent assay is another method in which plates coated with specific antibodies can be used to detect certain PTMs [25]. Immunoprecipitation combined with MS analysis is another method to detect PTMs. In general, antibody-based methods have a number of intrinsic limitations: detection is fully dependent on the availability of a specific and limited number of antibodies, which have limited analytical throughput and are suitable for the detection of predetermined PTMs.

**Figure 1:**
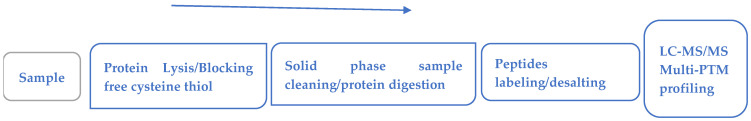
A simplified representation of the main components of a workflow for multi-PTM profiling: following lysis and the blockage of free cysteine thiols using N-ethylmaleimide, samples are automatically processed through the use of solid-phase beads. The resulting peptides are then labeled with tandem mass tag (TMT) reagents, conducted in a fashion that allows immediate chromatographic desalting. The pooled samples are split prior to protein quantification, followed by downstream enrichment to quantify cysteine thiol oxidation, phosphorylation, and acetylation. Figure 1 is based on material from Ref. [30] with thanks.

Over the last two decades, mass-spectrometry-based proteomics has emerged as the method of choice for the detection and structural characterization of PTMs. The increasing role of this technique in this particular area can be attributed to a number of relatively recent developments: First, a new generation of mass spectrometers has been introduced, possessing unprecedented high resolution, high mass accuracy, and high sensitivity. Second, the introduction of optimized, novel ion fragmentation methods, such as electron transfer [26], electron capture [27], and photo dissociation [28], allows for milder and more efficient ion fragmentation compared to more traditional collision-induced dissociation (CID) [29]. The introduction of these methods contributed to an enhanced role of tandem mass spectrometry (MS/MS) in structural biology, including more accurate identification of various sites of PTMs. These hardware developments have been accompanied by highly sophisticated informatic tools, including advanced software containing powerful algorithms, and almost comprehensive protein databases. On the sample preparation side, there have been substantial improvements in sample cleaning procedures, storage, and protein labeling, which is essential for accurate protein quantification, particularly when multiple modifications are present within the investigated sample. Existing LC-MS/MS platforms are still difficult to automate to allow high-throughput analyses of these modifications. In a recent article, a research group described a workflow that they claim to be suitable for multiple PTM characterizations within the same sample [30]. The main components of this workflow are given in Figure 1; the legend of the same figure gives further details, which are not indicated in the scheme. The role of MS-based proteomics in clinical settings has been described and discussed in a number of recent reviews [31,32,33,34].

### 1.2. The Complex Relationship Between Cytokines and Immunotherapy

Naturally generated cytokines are a large family of small, secreted proteins that serve as intercellular signaling molecules. This large family includes interleukins, interferons, tumor necrosis factors, chemokines, and growth factors [35]. The relationship between this family of proteins and the immune system is rather complex to say the least. For a number of years, cytokines have been described as one of the main challenges to efficacious CAR T and CAR NK cell therapies. This main challenge is due to a serious adverse event known as cytokine release syndrome (CRS), provoked by high levels of expression of naturally occurring cytokines in patients receiving immune cell therapy [15,16].

**Figure 2:**
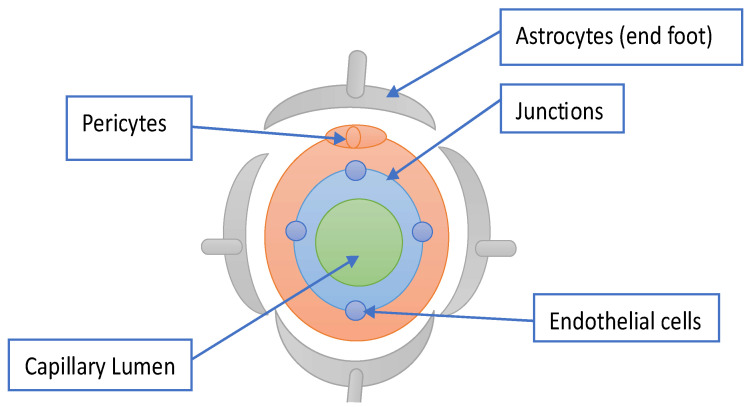
Schematic representation of the basic structure of blood–brain barrier (BBB). The main components in the scheme are endothelial cells, which are considered the core anatomical structure lining the cerebral blood vessels and interact with different types of cells in the central nervous system. Astrocytes are considered a type of neural cells that, together with pericytes, surround blood vessels in the brain, serving as the interface between neurons and endothelial cells. Pericytes are considered central to the proper function of the neurovascular unit. These mural cells are present at intervals along the walls of the capillary blood vessels. In the BBB, tight junctions are the main functional components in sustaining the permeability barrier and controlling tissue homeostasis. This scheme is partially based on data in Ref. [39] with thanks.

The success of CAR T cell therapy in treating hematological tumors is not matched by similar results for solid tumors, particularly brain cancers. There are a number of reasons behind such a disappointing outcome, including short persistence of the injected cells, limited infiltration capacity, and a suppressive tumor microenvironment [36]. In the case of brain tumors, an additional factor that reduces the efficacy of CAR T in treating brain cancers is the impermeability of blood–brain barrier (BBB), which prevents a wide range of chemotherapeutic agents from reaching the targeted areas. The impermeability of this barrier is considered one of the major drivers behind the resistance of different types of brain tumors. A further factor which contributes to such resistance is the presence of efflux transporter proteins within various zones of the brain [37,38]. The main structural components of the BBB are schematically depicted in Figure 2 [39].

In more recent years, synthetic/engineered cyclonites have been used to enhance the therapeutic capabilities of immune cell therapy. Various studies have reported different approaches in which synthetic and/or engineered cytokines are used to enhance the therapeutic capacity of immune cell therapy. Various studies have reported that CD19 CAR-T cells, supported with antigen-induced IL-15 expression, showed high rate of survival, expansion, and antitumor activity for Leukemia anti-lymphoma [40]. Other studies have also demonstrated that disialoganglioside (GD2) CAR T cells expressing IL-15 showed much improved infiltration and cytotoxicity in lung cancer, neuroblastoma, and glioblastoma in xenograft models [41,42,43]. This protein can contribute to CAR T cell survival, expansion and long-term persistence within the body. In a relatively recent study, it was demonstrated that IL-15 can efficiently activate CAR T cells to inhibit activation-induced cell death and increase the proportion of the T stem cell memory subpopulation [44]. In a relatively recent preclinical study, patients with relapsed or refractory large B-cell lymphoma were treated with engineered CD19-specific CAR-T cells to secrete IL-7 and CCL19 upon CD19 antigen engagement. The authors reported that such engineered cells demonstrated superior antitumor cytotoxicity compared to conventional anti-CD19 CAR-T cells [45].

Recent years have witnessed an increase in clinical trials to assess the efficacy of CAR T and NK cells armored with engineered or synthetic cytokines. Among the issues which some of these trials attempt to clarify is the impact of cytokines on persistence and the immunosuppressive tumor microenvironment (TME). Some of these trials are given in Table 1. All three trials were sponsored by Baylor College of Medicine, USA, and share similar objectives, indicated in the same table. The main objective of the three trials is to assess the efficacy of CAR T cells armored with interleukin(s) in the treatment of some types of solid tumors. More details on the methods used in the listed trials have been given in a recent investigation [45].

### 1.3. The Impact of Glycosylation on the Functions of Some Key Cytokines

There are over 650 types of protein post-translational modifications (PTMs), with over 400 formally cataloged in major biological databases [46,47]. Glycosylation is a post-translational modification where various carbohydrate chains get attached to various amino acids. In the case of IL-6, these chains can be linked to asparagine (N-glycans), to serine and threonine (O-glycans), or to tryptophan residues (C-glycans). The multiplicity of the modification sites of attachment, and the different configurations of the attached chains are responsible for the complex structural diversity of the resulting modified cytokines [17,48]. Glycosylation is known to play a key role in the regulation of protein function, cell adhesion and immune escape, activities are essential for cancer progression, tumor metastasis, and immune evasion [49,50]. To underline the impact of this PTM on the activities of some cytokines, some real-world examples are considered. Over 40 years ago, in a well-designed study, and using different analytical techniques, including metabolic labeling, glycosidase digestion, and lectin chromatography, the authors provided a very detailed picture on the glycosylation of IL-6 [48]. The same study reported that O-glycosylated species were observed in the molecular mass range 23–25 kDa, while those observed in the range 28–30 kDa were a mixture of both O- and N-glycosylated species. This study concluded that newly synthesized IL-6 polypeptides rapidly enter two separate protein modification pathways: the first is O-glycosylation and the second comprises both N- and O-glycosylation; both pathways can be further modified through phosphorylation prior to secretion. In another and more recent study [17], the authors emphasized that glycosylation affects the synthesis, receptor binding, signaling and plasma half-life of cytokines, as well as the protein stability, transport to the cell surface, ligand binding, proteolysis, internalization, and recycling of their receptors. This long list of functions, which can be influenced by glycosylation, needs to be supported by more robust proteomic, genetic and transcriptional data, which are not available yet. For example, there is a paucity of proteomic studies, which can provide much needed information on glycosylation and other PTMs, particularly on phosphorylation. The same studies can furnish vital information on the structure, conformation, complexation, and interaction of this protein with other proteins and with various ligands. Phosphorylation is another modification which IL-6 can experience. This modification targets serine and threonine residues, and is known to influence various biological functions of this protein, including its stability, signal transduction, transcriptional regulation, and intracellular localization [51].

## 2. Glycosylation of Some Immune Checkpoints and Its Impact on Their Therapeutic Roles

Currently, there is substantial evidence that underlines the important role of PTMS in the modulation of most known immune checkpoints. Such modulation is closely linked to the influence that some of these modifications exert on protein expression, localization, and interactions with other cellular molecules. Presently, there is a long list of immune checkpoints undergoing various clinical and preclinical investigations, yet only a few of them have been researched in depth to include the role of some PTMs in immunotherapy. In the present review, the impact of glycosylation on three checkpoints is discussed in Table 2.

### 2.1. Glycosylation of Programmed Cell Death-1 (PD-1) and Its Ligand (PD-L1)

Most known immune checkpoints can experience single and/or multiple PTMs. Glycosylation is one of the modifications known to stabilize these proteins, enhance their cell-surface localization, and facilitate their interactions with other cellular molecules. In recent years, N-glycosylation of PD-1 and PD-L1 has been the focus of numerous studies to clarify the impact of this modification on the role of both proteins in immunotherapy [55,56,57,58]. One of the questions which most of these studies try to address is the impact of this modification on the efficacy of immune cell inhibitors (ICIs). Understanding the mechanisms and the biology behind such a contribution is necessary for the design and implementation of new therapeutic strategies, which may lead to the development of new generation of ICIs capable of extending the therapeutic benefit of this therapy to a wider base of cancer patients.

The expression level of PD-L1 in tumor tissues in cancer patients is considered the main predictive biomarker for the stratification of patients who are likely to benefit from anti-PD-1/PD-L1 therapy. A number of studies have demonstrated that glycosylation can negatively influence the expression of this protein, which is commonly measured using immunohistochemical (IHC) staining. In other words, the presence of glycosylated PD-L1 renders its level of expression as a biomarker in certain types of cancer less reliable. This observation is supported by a work in which the removal of N-glycosylation enhanced PD-L1 detection, as well as the reliability of the test in assessing PD-1/PD-L1 inhibitors [13]. Present-day literature shows almost permanent interaction between the PD-1/PD-L1 pathway and PI3K/AKT/mTOR pathways, and such an interaction participates in the regulation of PD-L1 and in the activation of PI3K/AKT/mTOR [59,60]. The importance of this interaction to the treatment of some forms of cancer is reflected in some therapeutic strategies, in which the monoclonal antibody PD-1/PD-L1 was combined with PI3K/AKT/mTOR inhibitors for the treatment of NSCLC patients [59]. Glycosylation is the only PTM that has been investigated with some depth. This does not mean that other modifications have less impact on the interaction of the PD-1/PD-L1 pathway with other signaling pathways. These modifications include phosphorylation, methylation, and ubiquitination.

### 2.2. Therapeutic Strategies Based on PD-L1 Glycosylation

The level of expression of PD-L1 on cancer cells remains the main biomarker for the identification of the subset of patients who are likely to benefit most from PD-1/PD-L1 blockade. To enhance the detection of this checkpoint, and to improve its reliability as a biomarker, various studies have proposed deglycosylation prior to performing IHC staining to determine its level of expression [61]. The identification of reliable biomarkers for a given disease is central to any therapeutic strategy. For example, glycoproteins such as human epidermal growth factor receptor 2 (HER2) for breast cancer, alpha-fetoprotein (AFP) for liver cancer, and carcinoembryonic antigen (CEA) for colon cancer are among the biomarkers which are routinely used for the diagnosis and monitoring of the progression of the indicated diseases [62,63]. The PDL-1/PD-1 pathway has been the target of various investigations to establish its precise role in immune regulation and how such a role impacts on the efficacy of ICI therapy. It is well established that PD-L1 can experience glycosylation at four different asparagine sites (N35, N192, N200, and N219) [64]. From a structural point of view, these modifications are well characterized, however, their role in immune regulation, and how they influence the efficacy of ICIs remain to be fully clarified. For example, it is still not clear whether the functions of the modified protein are linked exclusively to a specific, single site or if they involve all four sites. A recent investigation of the N-glycosylation of PD-L1 and its impact on the efficacy of immune checkpoint blockades reported an interesting result: the N-glycosylation of PD-L1 at all four glycosylation sites interfered with the ability of ICI agents to block the interaction between PD-1 and PD-L1. In contrast, single glycosylation at N35 was found to enhance the blocking efficiency of these agents against the same reaction [56]. Another study illustrated that the glycoprofiling of surface receptors, together with their circulating counterparts, provided relevant information on the interaction of two monoclonal antibodies, amrelizumab and cemiplimab, with glycosylated PD-1. This study showed that the reaction of these antibodies with glycosylated PD-1 was highly selective, depending on the glycosylation form [65].

The last ten years have witnessed an unprecedented growth in the number of monoclonal antibodies (mAbs) targeting PD-1 and PD-L1. This growth is exemplified by the relatively fast approval by the FDA of ten mAbs for the treatment of a number of tumors. It is relevant to point out that such growth would not have been possible without the massive participation of big pharmaceutical companies. These antibodies have two main limitations. They are known to benefit a very restricted subset of patients. Furthermore, such limited number of patients cannot be identified prior to the application of the therapy due to the absence of reliable, predictive biomarkers. The absence of such biomarkers is behind the second main limitation of ICI therapy: such a limitation is due to serious immune-related adverse events (irAEs) provoked by the therapy itself, which in many cases necessitate the interruption of ICI therapy.

### 2.3. N-Glycosylation of Immune Checkpoint B7 Homolog 3 (B7-H3)

**Figure 3:**
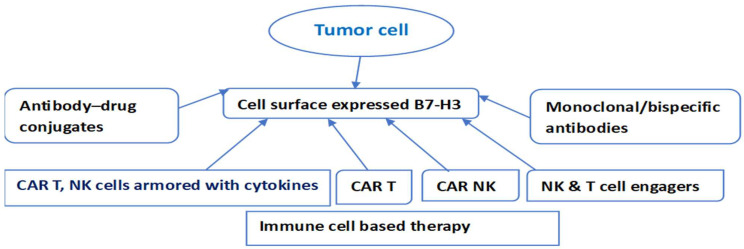
Different therapeutic modalities reported in different clinical trials and preclinical studies, targeting the B7-H3 immune checkpoint. Each of the indicated therapeutic approaches has its advantages and limitations. Currently, there are not sufficient clinical data to suggest that the contemporary application of more than one modality can influence the efficacy of the therapy.

Human B7-H3 is a member of the B7 family, which contains nine other members, including PD-L1 and PD-L2 [66,67]. This protein has two isomers, a 41 g isoform (human) and a minor isoform, 21 g (mouse) [67]. As well as phosphorylation and ubiquitination, B7-H3 is known to experience N-glycosylation at seven asparagine residues. The low expression of this protein in healthy tissues and its substantial expression, including its glycosylated form in various cancerous tissues, rendered this checkpoint a promising therapeutic target for various forms of cancer, in particular, solid tumors [52,54,68]. The potential of B7-H3 as a therapeutic target was tested in various clinical trials and indifferent preclinical studies. Such potential was tested using monoclonal antibodies, bispecific antibodies, and antibody–drug conjugates (ADCs) [69,70,71]. The poor results delivered by these therapeutic modalities paved the way to new cell-based therapeutic strategies, including chimeric antigen receptor (CAR T) and natural killer (NK) cells [60,72,73]. The various modalities to target B7-H3 are given in Figure 3.

### 2.4. Therapeutic Strategies Based on B7-H3 Glycosylation

There is clear experimental evidence that B7H3 is a highly N-glycosylated membrane protein. However, the key glycosylated asparagine residues that mediate the function of the B7H3 protein are still to be clarified. A relatively recent study reported that N-glycans attached to two pairs of asparagine residues, N91/309 and N104/32, are required for proper B7H3 localization on the cell surface membrane [53]. In this study, the authors suggested that targeting site-specific N-glycosylation of B7-H3 can represent a potential therapeutic strategy. Such a suggestion is based on two results within the same investigation. N-glycosylation at N91/309 and N104/322 of B7H3 is essential for its inhibition of T cell proliferation and activation. This result was enforced by data obtained using a monoclonal antibody developed by the same research group, Ab82. According to the authors, these investigations revealed that this monoclonal antibody selectively targets the same glycosylation sites, inducing B7H3 internalization and degradation, while enhancing antitumor immunity through cytotoxic T cell activation. It can be said that this study is rather unique, as understanding the glycosylation of B7-H3 at specific sites represents the central objective of the investigation. Non-modified B7-H3 has been a therapeutic target for numerous clinical trials (see Table 3). It is interesting to note that none of the listed trials refer to glycosylated B7-H3.

### 2.5. Examples Supporting the Role of PTMs in Various Forms of Cancer

Despite long years of research and numerous academic, clinical, and pharmaceutical investigations, the role of N-glycosylation in various diseases remains partially understood. The difficulty in deciphering its role is linked to the complex heterogeneity of N-glycosylation patterns (structures). In a relatively recent proteomic investigation, such complex heterogeneity has been clearly demonstrated [74]. The authors used LC-MS/MS and N-glycoproteomic analyses to examine cancerous tissues and adjacent normal tissues derived from the same glioblastoma patients. The N-glycosylation pattern was established through LC-MS/MS detection of intact glycopeptides. The same study identified and quantified over 1800 distinct intact glycopeptides with 161 N-linked glycan compositions and 326 glycosites. The same study reported that almost 400 glycopeptides from 43 glycoproteins were different between diseased tissues and their adjacent counterparts.

It is reasonably accepted that, are various mechanisms of glycosylation implicated in metastasis and tumor recurrence [75]. The current literature demonstrates the role of protein glycosylation in various cancer types. For example, over 20 years ago, it was reported that the fucosylation of E-cadherin caused diminished adhesion of lung cancer cells, promoting distant spread and recurrence [76]. Glycosylation of B7-H3 was found to stabilize and promote high B7-H3 expression in triple-negative breast cancer (TNBC) patients [68]. Pathways of some PTMs have been used to stratify glioma patients into various clusters. Inter-cluster differences in clinical outcomes, mutational landscapes, and the immune microenvironment were used with machine learning to construct a prognostic prediction model. *TOM1L1* was among the genes included in the model [77].

Oral squamous cell carcinoma is characterized by high morbidity and high rate of mortality, with limited therapeutic options. To identify new and more effective biomarkers for this form of cancer, the expression of glycosylated B7-H3 in both cancerous and normal cells was monitored [75]. This analysis revealed significantly upregulated B7-H3 in tumor tissues: such expression was found to be correlated with increased tumor size and a poor survival rate. The association of glycosylated proteins with various forms of cancer is well established. Many well-known tumor biomarkers, including alpha-fetoprotein (AFP), carcinoma embryonic antigen (CEA), CA125 and prostate-specific antigen (PSA), are glycosylated proteins [78,79,80,81]. The association of N-linked glycoproteins with postoperative relapse in hepatocellular carcinoma (HCC) patients was assessed [82]. A thousand serum samples taken from patients with early-stage HCC were examined, and identified glycoproteins were further validated through immunohistochemical staining of 193 HCC tissues. The authors concluded that a high level of core fucosylated quiescin sulfhydryl oxidase1 (cf-QSOX1) in the investigated serum samples was significantly associated with a longer time of disease recurrence after HCC resection. The same authors emphasized that the detection of QSOX1 in the investigated tumor tissues was associated with an encouraging prognosis in HCC patients. Univariate and multivariate analyses have also demonstrated that both serum cf-QSOX1 and tumor-expressed QSOX1 can be considered as independent prognostic indicators of the disease. It is fair to say that this study is very useful as a proof of concept, demonstrating that the levels of certain glycoproteins within patients’ serum and in tumor tissues can be used to monitor the recurrence of CCH. That said, the conclusions reported in this article need to be supported by further investigations involving higher numbers of subjects and higher number of samples, and, if possible, different detection methods.

## 3. Conclusions and Future Perspectives

There is almost a total agreement between the various research disciplines, academic, clinical, and pharmaceutical, that various protein post-translational modifications play a major role in cancer initiation and progression by influencing important biological processes such as protein folding, degradation, cellular localization, immune modulation, and intercellular signaling. Immune checkpoints represent an important component in the landscape of immunotherapy. Targeting this group of proteins with immune cell inhibitors represented one of the success stories in the world of oncology. Various members of this group of glycoproteins are known to experience different forms of PTMs, including extensive glycosylation. Limiting the present conclusions to the impact of some of these modifications on ongoing therapeutic strategies within immunotherapy, the following observations may shed some light on the current application of these modifications as part of current therapeutic strategies in immunotherapy: (a) Despite their recognized role in multiple biological processes, most PTMs remain understudied. This is particularly evident in the case of immune checkpoints; the current literature shows that a very limited number of modifications associated with few checkpoints have been investigated with some depth so far. (b) Major efforts in the landscape of immune therapy, such as the development of immune cell therapy and the search for innovative classes of antibodies, are based on the inhibition of certain protein expressions in various fatal tumors. That said, most of these research efforts seem to assume that most of the targeted proteins are intact (non-modified). The well-documented scientific literature tells another story, where hundreds of known PTMs are responsible for the complexity of the human proteome. Cellular localization of most known checkpoints has a direct impact on the extent of interaction between these proteins and their response to various therapeutic regimens. Given that one of the main biological functions of certain PTMs is their influence on protein localization, such modifications are bound to impact the efficacy of these regimens. The existing literature regarding the role of glycosylated checkpoints in immune therapy identifies a number of important limitations, which have to be addressed, before this class of modified proteins can fulfill its expected potential as highly promising immune therapeutic targets. One of the limitations is the absence of sufficient experimental knowledge of the relationship between checkpoint glycosylation and the therapeutic potential of these proteins. The absence of such knowledge is considered an obstacle on the path of translating glycosylation-based targets into clinical practice. That said, there are initial indications that such a limitation can be partially addressed through the wider application of glycoproteomics, glycoengineering, and the development of high-throughput workflows capable of the detection and quantification of multiple PTMs within clinical samples. It is generally agreed that glycosylation in cancer has functional consequences on the immune system; however, there are no drugs to directly target such a modification. Future developments in cancer glycomics and glycoproteomics are expected to address this gap by introducing new immunotherapies, specifically targeting modified checkpoints, which are known to impact the functionality of the immune system.

## Figures and Tables

**Table 1:** Some clinical trials to assess the efficacy of engineered CAR T cells armored with interlukin-15 in the treatment of some solid tumors.

Clinical Trials.gov ID/Sponsor	Description of the Trials
NCT05103631/Baylor College of Medicine, USA	This is a phase 1 study entitled “Interleukin-15 armored glypican-3-specific chimeric antigen receptor expressed in autologous T cells for solid tumors”. Study start: 2021-06; primary completion: 2025-01; study completion: 2040-01 (estimated); study type: interventional; actual number of patients enrolled: twelve. The main objective of this study is to establish the highest dose of CATCH T cells that is safe, to determine how long they last in the body, and to identify likely adverse events.
NCT02905188/Baylor College of Medicine, USA	This is a phase 1 study entitled “Glypican-3-specific chimeric antigen receptor expressing T Cells for Hepatocellular Carcinoma (GLYCAR)”. Study start: 2019-03; primary completion 2021-11; study completion 2023-01; study type: interventional; number of patients enrolled: Nine. The main objective of this study is to establish the highest safe dose of GLYCAR T cells, the duration of such dose within the patient, likely adverse events, and the therapeutic benefit of such treatment for patients suffering GPC3-positive hepatocellular carcinoma. The GLYCAR T cells are an investigational product not approved by the FDA.
NCT02932956/Baylor College of Medicine, USA	This is a phase1entitled “Glypican-3-specific chimeric antigen receptor expressed in T Cells for Patients with Pediatric Solid Tumors (GAP)”. Study start: 2018-12; primary completion: 2021-09; study completion: 2037-02 (estimated); study type: interventional; patients enrolled: Nine. The main objective of this study is to establish the highest safe dose of GAP T cells, the duration of such dose within patient’s body, adverse events associated with the therapy, and the therapeutic benefit for patients suffering GPC3-positive solid tumors (currently only enrolling liver tumors). The GAP T cells are an investigational product not approved by the FDA. Observations: GAP T cells are an experimental form of chimeric antigen receptor (CAR) T cell therapy designed to target tumors expressing the glypican-3 (GPC3) protein. Primarily investigated for solid tumors such as hepatocellular carcinoma (liver cancer), they work by genetically modifying a patient’s own T cells to recognize and attack GPC3-positive cancer cells. GLYCAR T cells (Glypican-3-specific chimeric antigen receptor expressing T Cells) are an experimental immunotherapy developed at Baylor College of Medicine and its associated centers. They are engineered to hunt down and destroy cancers that express the Glypican-3 (GPC3) protein. CATCH T cells are genetically engineered with a GPC3 CAR and IL15. CATCH T cells, they are investigational product not approved by the Food and Drug Administration (FDA). They are tested in patients having GPC3-positive solid tumors.

**Table 2:** Glycosylation at different asparagine (N) sites of PD-1, PD-L1, and B7-H3.

Immune Checkpoint	Glycosylation Sites/Ref.
PD-1	N49, N58, N74, N116 [52].
PD-L1	N35, N192, N200, N219 [13]
B7-H3	N91, N104, N189, N215, N309, N322, N407, 433 [53,54].

**Table 3:** Some ongoing clinical trials to assess the safety and efficacy of two therapeutic modalities, targeting B7-H3: CAR T cells and antibody–drug conjugates (I-DXd, YL201). All the therapies used in these trials are investigational, not approved by the FDA yet.

Clinical Trials.gov ID/Sponsor	Description of the Trial and Observations
NCT06347068/UNC Lineberger Comprehensive Cancer Center, North Carolina, USA	This is a phase 1, single-center, open-label study to investigate the safety of escalating doses of chimeric antigen receptor T cells (CAR T) in subjects with relapsed/refractory triple-negative breast cancer (TNBC). The study started in 2024 and is expected to be completed in 2028 (estimated).
CT05366179/UNC Lineberger Comprehensive Cancer Center, North Carolina, USA.	This is a phase 1, single-center, open-label study designed to assess the safety of escalating doses of chimeric antigen receptor T cells (CAR T), targeting the B7-H3 antigen administered via intraventricular infusion to adult subjects with relapsed or refractory glioblastoma (GBM). It started in 2022, and is expected to be completed in 2030.
NCT06612151/MediLink Therapeutics (Suzhou) Co., Ltd., Suzhou, China	A phase 3 study of YL201 in relapsed small-cell lung cancer. This study was designed to compare the efficacy and safety of YL201 with topotecan hydrochloride in subjects with relapsed small-cell lung cancer (SCLC). It started in 2024 and is expected to be completed in 2030 (estimated).
NCT06330064/Daiichi Sankyo, Inc., New Jersey, USA.	This is a phase 2 study, designed to assess the efficacy and safety of Ifinatamab Deruxtecan (I-DXd) in subjects with recurrent or metastatic solid tumors in patients previously treated with one or more systemic therapies for the selected tumor indication. It started in 2024, and will be completed in 2028 (estimated).
NCT06203210/Daiichi Sankyo, Inc., New Jersey, USA.	This is a phase 2 study; the primary objective of this study is to establish whether the treatment of small-cell lung cancer (SCLC) patients with I-DXd can improve the objective response rate (ORR) and prolong overall survival (OS) compared with treatment of physician’s choice. It started in 2024, and will be completed in 2029 (estimated).

## Data Availability

No new data were created or analyzed in this study. Data sharing is not applicable to this article.

## Abbreviations

CRS	Cytokine release syndrome
PTMs	Post-translational modifications
irAEs	Immune-related adverse events
MS/MS	Tandem mass spectrometry
LC-MS/MS	Liquid chromatography–tandem mass spectrometry
CID	Collision-induced dissociation
BBB	Blood–brain barrier
CAR-T	Chimeric antigen receptor
NK	Natural killer
GD2	Disialoganglioside
IHC	Immunohistochemical
PD-1	Programed cell death-1
PD-L1	Programmed cell death ligand
ICIs	Immune cell inhibitors
HER2	Human epidermal growth factor receptor 2
AFP	Alpha-fetoprotein
CEA	Carcinoembryonic antigen
FDA	Food and Drug Administration
SCLC	Small-cell lung cancer
